# Research “Factors affecting postpartum intention to use contraceptives in Wadajir, Mogadishu, Somalia”

**DOI:** 10.1186/s40834-026-00452-0

**Published:** 2026-05-18

**Authors:** Zahra Ali Farah, Timothy A. O. Oluwasola

**Affiliations:** 1 Mogadishu, Somalia; 2 Pan African University Life and Earth Sciences Institute (Including Health and Agriculture), University of Ibadan, Ibadan, Nigeria; 3 Gynaecology Oncology Unit Department of Obstetrics and Gynaecology College of Medicine, University of Ibadan, and University College Hospital, University of Ibadan, Ibadan, Nigeria

**Keywords:** Postpartum family planning, Contraceptive intention, Marital structure, Partner communication, Somalia

## Abstract

**Background:**

Family planning is typically given less priority by policymakers, service providers, and users. Family planning is widely acknowledged as a women’s and children’s health-improving and life-saving intervention. Due to the dearth of accurate and up-to-date national data on Somali women of reproductive age’s postpartum intention to use contraception, this research aimed to assess the factors affecting postpartum women’s intention to use contraceptives in Mogadishu, Somalia.

**Methods:**

This hospital-based descriptive cross-sectional study used quantitative data collection among 334 reproductive-aged women (19–49 years). Participants were selected by simple random sampling from two Wadajir health centers and Banadir Hospital. Women aged 19–49 years who were postpartum within the previous 12 months and who consented to participate were included. Women who delivered more than 12 months prior, were too ill to participate, or did not provide consent were excluded. Five trained research assistants collected the data. Quantitative data were coded, entered, and analyzed using IBM SPSS Statistics version 26. Descriptive statistics, frequencies, percentages, means, and medians were generated and presented in tables and graphs.

**Results:**

Of 334 women approached, 332 consented (response rate: 99.4%). The median age was 25.5 years (IQR: 23–31). Only 44.9% (95% CI: 39.5–50.3) had ever heard of modern contraceptives. Postpartum contraceptive use was reported by 37.7% of participants. The most commonly used methods were progestogen-only pills (22.4%) and combined oral contraceptives (18.4%). Despite low awareness, 87.3% expressed readiness to use modern contraceptives. Intention to use contraception was significantly associated with type of marital union and perceived importance of partner discussion.

**Conclusion:**

Awareness of modern contraceptives among postpartum women in Mogadishu remains limited; however, readiness to use them is high. Marital structure and partner communication play significant roles in contraceptive intention. Strengthening culturally sensitive counseling and promoting male involvement may improve postpartum contraceptive uptake in Somalia.

## Background

Post-partum family planning (PPFP) The World Health Organization defines preventing unexpected conception and successive pregnancies within the first year after following delivery [1]. Family planning is typically given less priority by policymakers, service providers, and users [2]. In addition, postpartum period is a time when women typically have a noteworthy unmet demand for FP [2]. Family planning is widely acknowledged as a women’s and children’s health-improving and life-saving intervention [3]. Moreover, women can start using a variety of safe and efficient contraceptive methods at different stages following delivery, including those used immediate postpartum, to maximize birth spacing [4]. By helping to avoid high-risk pregnancies, abortions, and unwanted pregnancies, FP has improved the well-being of families and communities. Globally, FP has the potential to eradicate hunger and poverty in nations with a high rate of conception, as well as avert 32% of all maternal fatalities and over 10% of all child fatalities [5]. Mothers receive a lot of standard interventions throughout the postpartum period. In sub-Saharan Africa, uptake of PPFP is still low while little is known about the factors that influence pregnant women’s decisions to use PPFP. However, the advantages of early embracing of, or continuing, FP are positive and well documented because factors such as breastfeeding, child care, menstrual resumption, and resumption of sexual relations make the postpartum period to be tough for women [6]. According to a survey conducted in 21 nations with low and moderate incomes, (LMICs), 90% of new mothers planned to use contraception in the future [7]. In USA, an assessed 91% of women reported intending to utilize modern contraceptive techniques [7]. Characteristics associated with the intention to use contraception include maternal age, use of antenatal care services, prior use of any form of contraception, and knowledge that at least one technique had been identified [8, 9]. In comparison to high-income nations, the usage of modern contraceptives is still relatively low in the LMICs. The low use of Intrauterine devices (IUDs) in the LMICs is a result of misunderstandings and misperceptions about long-acting modern contraceptives [10].

Since maternal mortality is excessively high in emergency-affected nations, PPFP has the potential to forestall more than 30% of maternal deaths by strategically spacing pregnancies [11]. Many women are unable to return for their 6-week postpartum visits in humanitarian settings, where accessing functional facilities is difficult and security risks limit movement. As a result, they are unable to receive FP counselling and adopt a method that is appropriate for their fertility intentions [12]. Uterine contractions after birth remove residual placental tissues and blood clots, and they might also do the same for any foreign body that was inserted into the womb. IUCDs placed after delivery had a significantly reduced danger of expulsion than those placed later in the postpartum phase, however, the rate of expulsion remains higher than that of interval insertions [11].

In order to improve mother and child health outcomes, the WHO technical consultative group recommends waiting for a minimum of two years following childbirth before becoming pregnant again [13]. Pregnancies that occur within a year of the mother’s previous birth are worse for the health of the mother and the child than those that occur at longer intervals. Children born within a year of a previous birth have a higher chance of death than those born at longer intervals [3]. Additionally, a higher risk of chronic under nutrition, stunted growth, and infant mortality is linked to closely spaced deliveries [13].

Therefore, it is a public health goal to reduce maternal mortalities and prevent unplanned and early pregnancies. Maternal and child mortality could be decreased by 30% and 10%, respectively, if couples used contemporary contraceptives to space out their pregnancies by at least two years [14]. Through education on contraceptive methods beginning early in pregnancy and continuing into the postpartum period, this can be scaled up. Poor use of postpartum contraception has been linked to a number of factors. These include the need to get pregnant soon, the worry about adverse effects, the inability to discontinue the procedure without consulting a doctor, the lack of understanding about the method, and the accessibility of the methods [14]. Furthermore, there are misconceptions, non-accessibility/poor accessibility for the procedure, and poor knowledge of current contraceptive insertions among healthcare professionals [15]. The benefits of contraceptive use go beyond the health sector as unrestricted access to contraceptives will guarantee fewer unplanned births, which will support female empowerment, increase female education, combat poverty, and even ensure environmental sustainability [15].

In Somalia, the total fertility rate, is 6.9 children for every woman. The TFR is lowest for city dwellers and higher for women who live in nomadic cultures, childbearing peaks between the ages of 20 and 29 and sharply declines after age 39. The General Fertility Rate in Somalia is 228 per 1,000 women on average. In Somalia, women who live in rural areas have a birth rate of 235 per 1,000, women who live in urban areas have a birth rate of 211 per 1,000, and women who live in nomadic households have a birth rate of 244 per 1,000. The TFR and GFR all display the same pattern, The TFR recorded in the Multiple Indicator Cluster Survey, (MICS) 2006, which each mother had 6.7 children, and the Somalia Health and demographic Survey, SHDS 2020, which reported 6.9 children per woman, disagree slightly. It is crucial to remember that the nomadic population was not included in the MICS 2006 coverage when comparing the two numbers. According to data from the SHDS, Somalia’s fertility rates have stayed largely consistent over the past two decades and have not decreased as predicted by the foreign experts [16].

The most popular technique was the combined oral contraceptives, then injected contraceptives that just contained progestin, while condoms were the least popular (Somali Health and Demographic Survey 2020) [17].

In comparison to health center’s maternal and child health (47%), hospitals provided more birth spacing services (52%), while governmental facilities outperformed the private sector (31% vs. 21%) [16].

The provision of checklists and standards for birth spacing was mandated for the healthcare facilities in order to consistently evaluate the degree of service delivery [17].

The Strategic and Action Plan for Reproductive Health in Somalia, 2010–2015 prioritized use and access to FP services. However, the likelihood of offering PPFP is further limited because, despite this priority, more than 90% of women continue to give birth at home and more than half receive assistance from a traditional birth attendant [17]. WHO, UNICEF, and other Aid agencies active in Somalia have emphasized the country’s poor access to competent delivery care and emergency obstetric care. Nurses and midwives who are trained to administer contraceptive methods like short-acting, natural methods are only found in a small number of health facilities that are supported by FP programs. These training’s though, are few and inconsistent. Despite these difficulties, it is crucial to provide postnatal FP counselling to women, including information on postnatal IUDs and implants, and to work towards making a full spectrum of postnatal contraceptive methods accessible [18].

### Research objectives

1. To determine the awareness of postpartum usage of contraceptives among the respondents.
2. To evaluate the readiness of postpartum women in using contraceptives.
3. To identify barriers and facilitators of postpartum contraception uptake.

### Research questions

1. What is the level of awareness of postpartum usage of contraceptives among the respondents in Mogadishu, Somalia?
2. How ready are postpartum women in using contraceptives?
3. What are the barriers and facilitators of postpartum contraception uptake among the respondents?

## Methods

### Study setting

This study was carried out in Banadir main hospital and two other health centers situated in Somalia’s Banadir region, in Mogadishu. In the Horn of Africa sits the nation of Somalia. Ethiopia borders it on the west, Djibouti to the northwest, Kenya to the southwest, the Indian Ocean to the east, and the Gulf of Aden to the north.

Banadir Hospital is a hospital for mothers and children as well as a national referral hospital. In 1976, the hospital was built as a part of a bigger set of facilities as part of a Chinese government development project for Somalis. Although there are 700 beds at the hospital, only 550 of them are being used at the moment. It is overseen by the Minister of Health (FMOH). 398 people work there overall, counting non-medical staff. About 3,000,000 people are served by the hospital in Mogadishu and the surrounding areas. The perimeter (area) of the hospital is 300 m^2^, of which 300 m × 250 m are under construction and 300 m × 50 m remained underdeveloped. Over three thousand people utilize Banadir.

### Study population and sample

The data were collected from women accessing care in the selected health facilities (Banadir hospital and 2 other selected health centers) which provide post-natal care and vaccination services in Mogadishu.

### Sampling techniques

Participants were chosen with through simple random sampling from two Wadajir health centers and Banadir Hospital.

**Eligibility criteria**

**Inclusion criteria**

In the study, the following women were included:

1. Women aged between 19 and 49 years.
2. Postpartum women who gave birth within 12 months as at the time of the study.
3. Women who consent to participate in the study.

### Exclusion criteria

The following women were excluded:

1. Women who gave birth 12 months prior at the time of the study.
2. Too ill to participate.
3. Refusal to consent.

### Sample size determination

The formula proposed by Andrew Fisher was used to calculate the sample size.

The smallest sample size for the population that will provide an expected level of assurance in the answers to the study questions was estimated using the formula [19].

$${\rm{n = }}{{{{\rm{Z}}^{\rm{2}}}{\rm{pq}}} \over {{{\rm{d}}^{\rm{2}}}}}$$

Where;

**n =** the desired sample size.

**z**= Confidence interval at 95% set as 1.96.

p = the prevalence of postpartum modern contraceptive uptake was 27% in Ethiopia (23) or 0.27.

**q** = the difference between 1 and *p* = 73% or 0.73.

**d**= maximum error allowed (5%, if the confidence level is 95%); 0.05.

Substituting into the formula, the sample size was computed as follows;

$$\:n=\frac{\left(1.96\right){\:}^{2}\:x\:0.27\times\:0.73\:}{\left(0.05\right){\:}^{2}}=\:302.9\:=\hspace{0.17em}303$$

Assuming 10% of non-responsiveness rate, the final sample size was:

[(0.10*303) + 303] = **333.2 women = 334 participants**

### Instrument for data collection

A total number of 334 structured self-reported questionnaire were distrubted.

Data were collected using a structured interviewer-administered questionnaire consisting of three sections:

1. Socio-demographic characteristics.
2. Obstetric and clinical characteristics.
3. Postpartum contraception knowledge, use, and intention.

### Validity

The design and pre-testing of the questions most likely guaranteed the validity of the study instruments. To guarantee the quality, research assistants were trained on how to administer the questionnaire.

### Reliability

Thirty or 10% of the questionnaires were pretested at Mogadishu’s randomly chosen prenatal care facilities for pilot study, should be done on 10% of the sample size.

### Modifications made to the tool after the pilot study

Ambiguous questions were reworded to enhance clarity and minimize the risk of misinterpretation. Complex terms were simplified to ensure that respondents could easily understand the items. The order of certain questions was adjusted to improve the logical flow, and repetitive items were removed.

### Data collection procedure

Five research assistants were recruited and trained on data collection, training was conducted for four consecutive days to be the principal of the investigator. The five nurses who are proficient Somali nationals, and a standardized and pretested questionnaire were used in the face-to-face interview method used to gather the data, every questionnaire was examined before distribution to guarantee accuracy and comprehensiveness of the information.

Data collection period was 26th March to 30th May 2023 in selected health facilities of Mogadishu.

### Analytical measures

The lead investigator gathered the completed questionnaire copies under the guidance of study supervisors and with assistance from a professional biostatistician, the research team performed data cleaning, coding, entry, and analysis. IBM SPSS version 26 was used to code, enter, and analyze each questionnaire. There was use of descriptive statistics. When necessary, frequencies, percentages, means, and medians were calculated and displayed as tables and graphs to illustrate particular demographic traits. A 5% (0.05) P-value was chosen, and confidence intervals were also be computed.

## Results

For this study, 334 women who were seeking post-natal care were approached and of these, the study was approved by 332 women, yielding a 99.4% response rate. Women’s age ranged from 19 to 45 with a median age (interquartile range) of 25.5 (23–31) years, and more than two-fifths of all respondents (137; 41.3%) belonged to the age group that spanned from 19 to 24 years. Majority, 234 (70.5%), of the study participants were in an intact form of marital status, with 233 (70.2%) being in monogamous form of relationship 122 (36.7%) reported to have attended to primary school while a relatively lower proportion 71 (21.4%) of women stated that their partners attended up to primary school (Table 1).

With regard to occupational category (105; 31.6%) of the women were unemployed while (74; 22.3%) of their partners were self-employed. Moreover 69 (20.8%) of the women were housemaids whereas sixty-four (19.3%) had partners who were farmers. Half of the respondents (166) described that their main source of income was their partner (Table 1).

Table 1 summarized the study variables, including the dependent variable and independent variables along with their respective categories.

**Table 1:** Awareness, magnitude of use, and readiness to use profiles

Variable	Frequency	Percent (%)
**Age group**		
19–24 years	137	41.3
25–29 years	102	30.7
30–34 years	51	15.4
35–39 years	23	6.9
≥ 40 years	19	5.7
**Current marital status**		
Married	234	70.5
Divorced/separated	55	16.6
Widowed	43	13.0
**Type of marriage**		
Monogamous	233	70.2
Polygamous	99	29.8
**Educational status**		
Non-formal education	138	41.6
Primary education	122	36.7
Secondary education	47	14.2
College diploma +	25	7.5
**Partner’s educational status**		
Non-formal education	135	40.7
Primary education	71	21.4
Secondary education	61	18.4
College diploma +	65	19.6
**Occupational category**		
Unemployed	105	31.6
Housemaid	69	20.8
Self-employed	44	13.3
Farmer	40	12.0
Student	36	10.8
Employed	29	8.7
Other	9	2.9
**Partner’s occupational category**		
Self-employed	74	22.3
Farmer	64	19.3
Employed	50	15.1
Student	37	11.1
Daily laborer	39	11.7
Unemployed	68	20.5
**Source of income**		
Partner	166	50.0
Parents	90	27.1
Relative	76	22.9

Regarding the place of delivery, the most commonly cited one was home with a traditional birth attendant 92 (27.7%), then followed by Mother and Child Hospital 60 (18.1%) and health center 58 (17.5%). Majority 188 (56.6%) of the women gave birth to term infants. About half 146 (44%) of the women claimed to had been pregnant 4 to 6 times before the time of interview. Moreover, nearly half 158 (47.6%) had one to three children. Two-thirds 220 (66.3%) of the women had spontaneous vaginal delivery of the index child (Table 2).

Meanwhile, 227 (68.4%) of the women had bad obstetric history. More than a quarter 60 (26.4%) of these women had preeclampsia/eclampsia while premature labor complicated 50 (22%) of these deliveries. Finally, most 322 (97%) of the women had children younger than a year by the time of interview (Table 2).

**Table 2:** Women’s reproductive traits seeking postnatal care from 26th March to 30th May 2023 in selected health facilities of Mogadishu, Somalia (*n* = 332)

Variable	Frequency	Percent (%)
**Place of delivery**		
Traditional birth attendant	92	27.7
Mother and Child Hospital	60	18.1
Health center	58	17.5
National hospital	47	14.2
District hospital	46	13.9
Regional hospital	29	8.7
**Gestational age at delivery**		
< 28 weeks	62	18.7
28–37 weeks	46	13.9
37–42 weeks	188	56.6
> 42 weeks	36	10.8
**Gravidity**		
< 4 times	53	16.0
4–6 times	146	44.0
7–9 times	89	26.8
≥ 10 times	44	13.3
**Number of live children**		
1–3	158	47.6
4–6	120	36.1
7–9	39	11.7
> 9	15	4.5
**Mode of delivery**		
Spontaneous vaginal delivery	220	66.3
Instrumental	62	18.7
Caesarean section	50	15.0
**Bad obstetric history**		
Yes	227	68.4
No	105	31.6
**Type of obstetric complication (** ***n*** ** = 227)**		
Preeclampsia/eclampsia	60	26.4
Premature labor	50	22.0
Placenta praevia	40	17.6
Placental abruption	36	3.5
Other	41	18.1
**Under 12 months child**		
Yes	322	97.0
No	10	3.0

Majority 241 (72.6%) of the women claimed to resume sexual intercourse within months following the last delivery. Only 149 (44.9%) ever heard of contraceptives. The two most commonly mentioned source of information were radio and healthcare workers, which were observed in 46 (30.9%) and 45 (30.2%) of the women, respectively. More than three-fourths 264 (79.5%) acknowledge the importance of discussion with their respective partner. 254 (76.5%) described to have interest to know about modern contraceptives. Most 272 (81.9%) acknowledged the importance of using contraceptives. Finally, more than two-thirds 224 (67.5%) of the women stated that they were very likely to be pregnant in the next twelve months (Table 3).

**Table 3:** Contraception related characteristics of women seeking postnatal care from 26th March to 30th May in selected health facilities of Mogadishu, Somalia (*n* = 332)

Variable	Frequency	Percent (%)
**Resume of sex**		
Within weeks	91	27.4
Within months	241	72.6
**Ever heard of contraceptives**		
Yes	149	44.9
No	183	55.1
**Source of information (** ***n*** ** = 149)**		
Radio	46	30.9
Healthcare workers	45	30.2
Peers	37	24.8
Television	21	14.1
**Source of contraceptive device (** ***n*** ** = 132)**		
Hospital	38	28.8
Health Center	57	43.2
Mother & Child Center	28	21.2
Private Clinic	9	6.8
**Types of contraceptive methods (** ***n*** ** = 125)**		
Progestogen-Only Pill	28	22.4
Combination pills	23	18.4
Implant	20	16.0
Condom	16	12.8
Natural method	12	9.6
Emergency contraceptive	10	8.0
Intrauterine device	8	6.4
DMPA	5	4.0
Calendar method	3	2.4
**Perceived importance of discussion**		
Yes	264	79.5
No	68	20.5
**Interest to know**		
Yes	254	76.5
No	78	23.5
**Perceived importance of use**		
Yes	272	81.9
No	60	18.1
**Future reproductive wish**		
Very likely	224	67.5
Somewhat likely	76	22.9
Unlikely	32	9.6

### Awareness, magnitude of use and readiness to use postpartum contraception

**Fig. 1:**
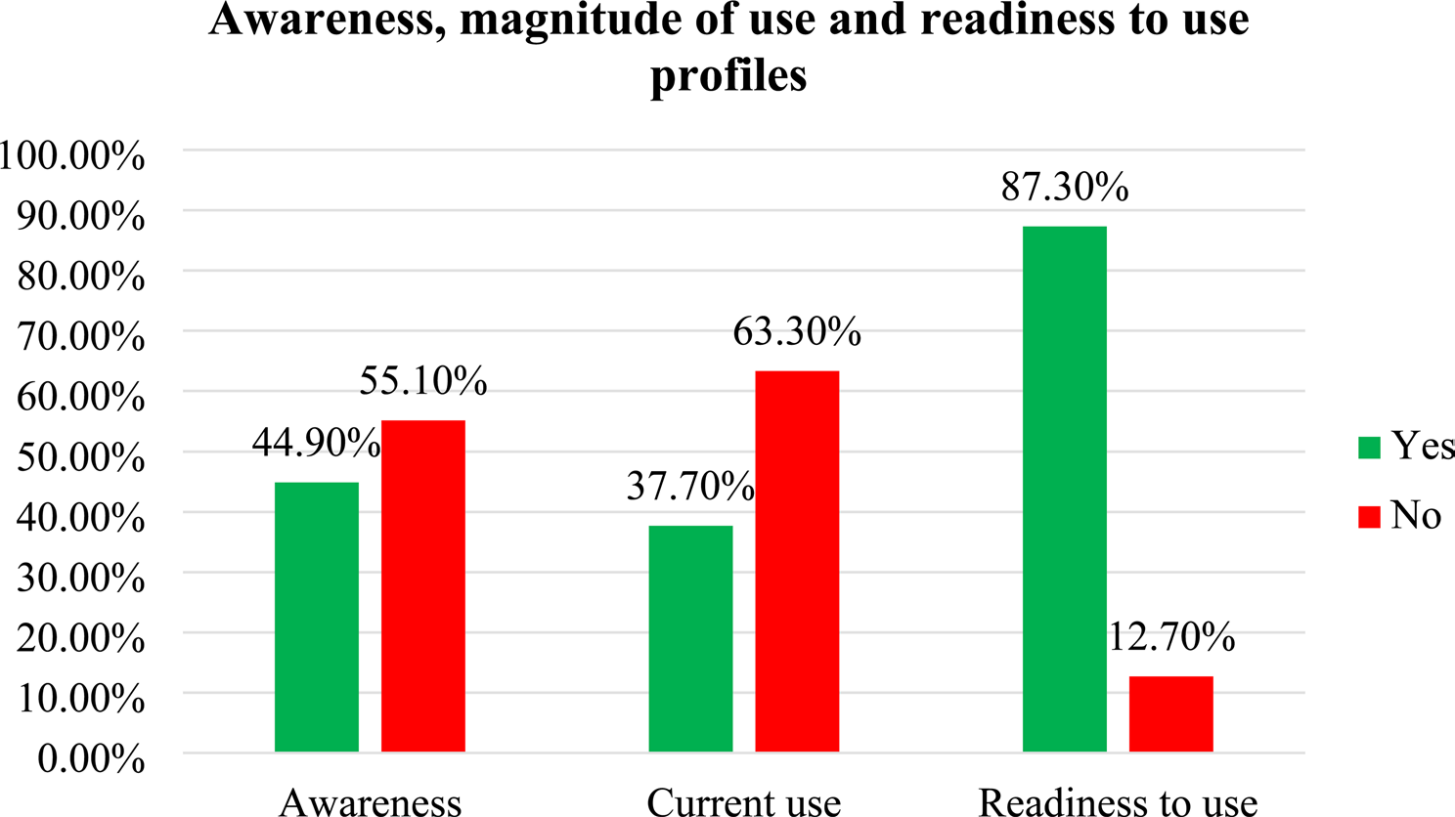
Awareness, magnitude of use and readiness to use profiles of women seeking postnatal care from 26th March to 30th May in selected health facilities of Mogadishu, Somalia

Among the women included in this study, 149 (44.9%; 95%CI: 39.5–50.3) had ever heard of modern contraceptives by the time of interview. 225(37.7%) were using some form of contraceptive methods following the index birth while the remaining majority 207 (62.3%) did not use any form of modern contraceptive. According to this study, the most popular methods of contraception were progestogen-only pills and combination pills, which were mentioned by 28 (22.4%) and 23 (18.4%) of the women respectively. Most 290 (87.3%) of the women were ready to use the available modern contraceptive methods (Fig. 1).

### Factors associated with postpartum intention to use contraceptives

In the present study, nineteen potential variables, namely age group, current marital status, type of marriage, educational status, partner’s educational status, occupational category, partner’s occupational category, source of income, place of delivery, gestational age at delivery, gravidity, number of live children, mode of delivery, bad obstetric history, under 12 months child, ever heard of contraceptives, timing of resume of sex, perceived importance of discussion and current use of contraceptives were incorporated into the study of regression. To ascertain the contributing elements to postpartum intention to use contraceptives while controlling possible confounders, adjusted odds ratios were produced by exporting independent variables with a p-value of less than 0.25 from the binary logistic regression to a multiple regression model.

Following this, only seven factors, namely type of marriage, place of delivery, gestational age at delivery, number of live children, ever heard of contraceptives, perceived importance of discussion and after a binary logistic regression revealed that the current usage of contraceptives had a p value of less than 0.25, the data was exported to a multiple logistic regression.

Enter method was applied to enter variables in the multivariate analysis. Thus, the only variables exhibiting a statistically significant correlation with women’s intention to use FP were type of marriage and perceived importance of discussion with a partner (Table 4).

More specifically, this study showed that, when compared to parents who stated to be in a polygamous marital relationship, those who reported to be in a monogamous marital relationship were more likely [AOR = 2.60 (95%CI: 1.16, 5.82)] to have postpartum intention to use contraceptives. Similarly, those who perceive that discussing contraceptives with a partner is important were more likely to have postpartum intention to use contraceptives [AOR (95%CI):7.65 (3.47, 16.84)] in comparison to their counterparts who do not claim that discussing contraceptives with a partner is important (Table 4).

**Table 4:** Factors associated with postpartum intention to use contraceptives among women seeking postnatal care in selected health facilities of Mogadishu, Somalia

Variable	Intention to use		COR(95%CI)	AOR(95%CI)
	Yes (%)	No (%)		
**Type of marriage**				
Monogamy	208	25	1.72(0.88,3.36)	**2.60(1.16**,**5.82)**
Polygamy	82	17	1	1
**Gestational age at delivery**				
< 28 weeks	51	11	0.92(0.31,2.76)	0.73(0.20,2.62)
28–37 weeks	38	8	0.95(0.30,3.04)	0.93(0.24,3.65)
37–42 weeks	171	17	2.01(0.73,5.51)	2.34(0.73,7.49)
> 42 weeks	30	6	1	1
**Place of delivery**				
Mother and Child Hospital	53	7	2.10(0.83,5.34)	1.29(0.44,3.76)
Health center	52	6	2.41(0.91,6.41)	1.67(0.53,5.26)
District hospital	43	3	3.98(1.12,14.19)	2.50(0.60,10.36)
Regional hospital	27	2	3.75(0.82,17.14)	3.25(0.60,17.68)
National hospital	43	4	2.99(0.96,9.32)	1.46(0.42,5.047)
Traditional birth attendant	72	20	1	1
**Ever heard of contraceptives**				
Yes	136	13	1.97(0.98,3.94)	3.50(0.41,29.76)
No	154	29	1	1
**Number of live children**				
1–3	132	26	0.78(0.17,3.67)	1.02(0.19,5.59)
4–6	112	8	2.15(0.41,11.24)	2.95(0.47,18.31)
7–9	33	6	0.85(0.15,4.74)	0.71(0.11,4.74)
> 9	13	2	1	1
**Current use of contraceptives**				
Yes	113	12	1	1
No	177	30	0.63(0.31,1.27)	3.86(0.43,34.22)
**Importance of discussion**				
Yes	246	18	7.46(3.74,14.86)	**7.65(3.47**,**16.84)**
No	44	24	1	1

Only factors which showed p value of < 0.25 in binary regression are shown here

Outcomes with statistically significant p values are in bold; 1: Reference category

## Discussion

The purpose of this study was to evaluate postpartum awareness and preparation, as well as the use of contraceptives among postpartum women, while identifying the factors affecting postpartum women’s intention to use contraceptives in Mogadishu, Somalia. Consequently, it revealed that close to half (44.9%) were aware of modern contraceptives. Most (87%) indicated readiness to use modern contraceptives. Further, it was demonstrated that women’s intention to use FP was affected by the type of marital union and perceived importance of discussion with a partner in Somali setting.

In the present study, only about half (44.9%) were aware of modern contraceptives. This is close to the work of Olajide et al., which documented that only about two-fifths of Nigerian women (38%) had ever heard about modern contraceptives (21). Conversely, the current result is far less than the finding observed in Tanzania, where almost all of the women (99.1%) had heard about modern contraceptives [20]. Likewise, the current finding was markedly different from another Nigerian report, in which majority (90.7%) had ever heard about modern contraceptives [21].

The low level of awareness can be attributed to the unique sociocultural dynamics in developing settings such as Somalia and belief systems, including low women’s education level, misconceptions, and spirituality. More strikingly, Somali couples equate using modern contraception to intentional infertility, which is in contradiction with the Somali culture of reproduction [22, 23].

Most (87%) of the studied women indicated readiness to use modern contraceptives. This finding is comparable to the level of intention (84.3%) to use modern contraceptive methods in Northern Ethiopia [8]. This result is slightly different from the finding observed in United State of America, where most women (91%) intended to use contraception [24]. On the other hand, the current study was comparatively higher than the Ethiopian study, which demonstrated that 70% of postpartum women had an intention on modern contraceptive use [25]. Again, it was much more than the figure obtained by Daba et al., who showed that 34.9% of pregnant women intended to use postpartum contraceptives [11]. This variance may be accounted for by the differences of studied population, study setting, gap in study period, accesses to information and availability to services use. Regarding the factors affecting the readiness to use modern contraceptives, women in monogamous marital relationship were more prone to intend to use modern contraceptives compared to those in polygamy.

This is in keeping with the work of Greenbaum and Bitire which highlighted that women who are or were in polygamous unions were less likely to use any contraceptive method compared to women who are or were in monogamous unions [26].

There could be various reasons for women in monogamous marital unions to be more likely to use contraceptives than women in polygamous union. One of the reasons include the possibility that women in polygamous unions may feel pressure to have more children than their co-wives to win their husband’s favor, leading to a lower use of modern contraceptives. Besides, women in polygamous unions may have less economic power and decision-making authority than women in monogamous unions, which can affect their ability to access and use of modern contraceptive [25]. Again, women in polygamous relationship can have less frequent sexual contacts in comparison to women in monogamous marital unions, making them in lesser need of contraception. This study found a high correlation between partners’ willingness or approval of contraception and women’s intention to use contraceptive techniques throughout the postpartum period. This finding is in line with another study conducted in Southern Ethiopia [25], which found that women intending to take contraception were more likely to get their husbands’ support. This could be the case since any factor affecting the partner’s attitude toward contraceptives would have an impact on women’s use of postpartum contraceptives [20].

## Strengths and limitations

### Strengths

- This study used quantitative research methods to capitalize on the strengths by providing stronger evidence and more confidence in the findings.
- Data were collected via face-to-face interviews, with the intention of reducing the chance of misunderstanding the questions.

### Limitations of the study

This study has several limitations. **First**, the cross-sectional design precludes the establishment of causal relationships between variables cannot be interpreted as evidence of causality.

**Second**, data on contraceptive use and non-use were obtained through participant self-report. Self-reported information is subject to reporting bias and may be influenced by underreporting, potentially leading to underestimated prevalence estimates. To reduce this risk, research assistants were carefully trained in standardized techniques for collecting sensitive information to enhance reporting accuracy.

**Third**, social desirability bias may have affected responses, particularly on culturally sensitive issues, as participants might have provided answers perceived to be socially acceptable rather than strictly truthful. Finally, the study was conducted exclusively in Mogadishu, which may limit the generalizability of the findings to other regions of Somalia where socio-cultural and contextual factors related to contraceptive use may differ.

### Recommendations

#### For healthcare professionals

Healthcare providers should strengthen education on postpartum modern contraceptive methods during both antenatal and postnatal care to improve awareness and reduce the incidence of unintended pregnancies.

#### For policymakers and regulatory bodies

The Ministry of Health, in partnership with local health organizations and other stakeholders, should implement targeted educational interventions for women of reproductive age to enhance knowledge about modern contraception and promote women’s empowerment through basic education. Community leaders should receive training on modern contraception to help foster positive community attitudes and support uptake.

#### For researchers

Future studies are encouraged to validate the current findings. Research that focuses on community-level dynamics and influences will contribute to a deeper understanding of factors affecting contraceptive uptake and inform more effective interventions.

## Conclussions

The findings suggest that while awareness and overall contraceptive use are relatively high, the adoption of modern contraceptive methods remains limited in the Somali context. Fear of side effects leads many women to rely on less effective traditional methods. Women’s intention to use family planning is influenced by marital structure and the importance of partner communication.

Marital structure and partner communication play significant roles in contraceptive intention. Tailored health education and culturally sensitive counseling strategies are recommended to improve the uptake of modern contraceptives in similar settings.

Strengthening culturally sensitive counseling and promoting male involvement may improve postpartum contraceptive uptake in Somalia.

## Data Availability

The data that support the findings of this study are available from the authors but restrictions apply to the availability of these data and are not publicly available.Data are however available from the authors upon reasonable request.

## Abbreviations

SPSS: Statistical Package for the Social Sciences
AOR: Adjusted odds ratio
PPFP: Post-partum family planning
FP: Family planning
LMICs: Low and middle income countries
IUDs: Intrauterine devices
WHO: World health organization
TFR: Total fertility rate
MICS: Multiple Indicator Cluster Survey
SHDS: Somalia Health and demographic Survey
MCH: Maternal and child health
